# Aucubin ameliorates diabetic kidney disease by restoring hGENCs autophagy through promoting phosphorylation of ATG4B protein

**DOI:** 10.1080/0886022X.2025.2605756

**Published:** 2026-01-14

**Authors:** Hong Wang, Lei Yang, Dishu Ao, Kai Yang, Songzhu Zou, Zhengjun Lin, Qi Liu, Kunming Wen

**Affiliations:** a Department of Nephrorheumatology, Soochow University Medical College, Suzhou, Jiangsu, China; b Department of Nephrorheumatology, The Second Affiliated Hospital of Zunyi Medical University, Zunyi, Guizhou, China; c Department of Microbiology, School of Basic Medical Sciences, Zunyi Medical University, Zunyi, Guizhou, China; d Department of General Surgery, Affiliated Hospital of Zunyi Medical University, Zunyi, Guizhou, China; e Department of Nephrorheumatology, Affiliated Hospital of Zunyi Medical University, Guizhou, China

**Keywords:** Aucubin, diabetic kidney disease, autophagy, ATG4B, phosphorylation, glomerular endothelial cells

## Abstract

Aucubin is a major component of *Eucommia ulmoides*, a traditional Chinese medicine used to treat diabetic kidney disease (DKD). However, the protective effect and mechanism of action of aucubin in DKD remains unclear. In this study, we found that aucubin decreased proteinuria in a DKD mouse model and alleviated human glomerular endothelial cells (hGENCs) damage caused by high glucose (HG). We labeled and quantified the total proteome and phosphorylated proteome of hGENCs using mass spectrometry, and the subsequent direct-data-independent acquisition analysis results showed that ATG4B protein phosphorylation is a prospective target of aucubin. We found that aucubin increased the phosphorylation level of ATG4B, restored autophagy, and weakened endothelial-mesenchymal transformation to protect against DKD *in vivo* and *in vitro*. Importantly, specific deletion of p-ATG4B aggravated HG-induced damage and eliminated the effects of aucubin-mediated protection in hGENCs. In conclusion, our study demonstrated that aucubin has protective effects against HG-induced hGENCs injury and in a DKD mouse model by upregulating p-ATG4B levels and restoring autophagy. This establishes p-ATG4B as a potential target for delaying DKD progression.

## Introduction

Approximately 20–40% of diabetic patients develop Diabetic Kidney Disease (DKD), which is the primary microvascular complication of diabetes [1,2]. The pathological manifestations of DKD include glomerular basement membrane thickening, mesangial hyperplasia, endothelial changes, and podocyte injury [3]. Clinically, DKD is often characterized by persistent proteinuria, elevated blood pressure, and reduced glomerular filtration rate [4]. Alarmingly, approximately 40–50% of DKD cases eventually progress to end-stage renal disease (ESRD), resulting in a huge global economic burden [1,5].

Although the currently available drugs for treating DKD have demonstrated some short-term effectiveness in controlling its progression, the underlying pathophysiology of DKD remains elusive, and there is a dire need for more effective therapeutics to prevent its progression to ESRD.

Due to the existence of the ‘metabolic memory’ phenomenon [6], the treatment of DKD, in addition to controlling blood sugar, also requires treatment targeting its pathogenesis. Early studies have found that the overactivity of renin-angiotensin-aldosterone (RAAS) is involved in the pathogenesis of DKD. Thus, in the past, the treatment of DKD mainly controls blood glucose and blood pressure using specific types of antihypertensive drugs that block RAAS [7]. This treatment has achieved certain effects in protecting the renal function of DKD patients. However, not all DKD patients can benefit from it, and some patients progress to ESRD.

In addition to strictly controlling blood sugar and using RAAS inhibitors to control blood pressure, the current clinical treatment drugs for DKD also include lipid-lowering drugs, probucol, DDD-4 inhibitors, pentoxifylline, and so on [8]. Since 2019, new drugs, such as sodium-glucose cotransporter 2 (SGLT2) inhibitors, have been used for the treatment of DKD. Clinical studies on CREDENCES have shown that SGLT2 inhibitors have achieved positive results in controlling DKD progression. However, during the short follow-up period, 5.27% of DKD patients (116 of 2,202) continued to progress to ESRD despite using both RAAS and SGLT2 inhibitors [9]. Therefore, for DKD patients who do not respond well to current treatment methods, new drugs need to be developed to slow down the progression of the disease [7].

Various mechanisms are involved in the onset and development of DKD, such as hemodynamic abnormalities, inflammatory responses, oxidative stress, endoplasmic reticulum stress, genetic and epigenetic dysregulation, and autophagy dysregulation [10–14]. Autophagy is a cellular process that plays a pivotal role in the breakdown and elimination of aging organelles and macromolecules, thereby enabling cells to eliminate waste, regenerate cellular components, harvest energy and nutrients, and maintain intracellular homeostasis. Previous studies have shown that dysregulation of autophagy can lead to the progression of DKD in diabetic hyperglycemia [15–18], whereas restoring autophagy has been shown to alleviate DKD [19–22].

As a traditional Chinese medicine, *Eucommia ulmoides* is widely used for the treatment of DKD [23–25]. Aucubin, with a molecular formula of C15H22O9, is an active ingredient extracted from *E. ulmoides*. Previous research has demonstrated that aucubin exhibits protective properties against various diseases such as neurological diseases, osteonecrosis of the femoral head, liver ischemia-reperfusion injury, hepatic dyslipidemia, traumatic brain injury, and diabetes. The mechanisms underlying these effects involve anti-inflammatory and antioxidant effects, regulation of endoplasmic reticulum stress, reduction of glycosylation end products, and regulation of autophagy [26–32], all of which contribute to alleviating DKD. While studies of aucubin in DKD are few, existing studies suggest that aucubin can alleviate inflammation, albuminuria, enlargement of the glomerular extracellular matrix, and renal fibrosis by inhibiting NF-κB signaling pathway activation and inducing the SIRT1/SIRT3-FOXO3a axis in diabetic nephropathy mice [33]. This study aimed to elucidate the effect of aucubin on DKD and explore its potential mechanisms. Our findings revealed that aucubin significantly alleviated renal function impairment in C57BL/6J mice with DKD and attenuated high glucose (HG)-induced human glomerular endothelial cells (hGENCs) injury. The underlying mechanism of these effects involves the restoration of autophagy by promoting the phosphorylation of ATG4B in hGENCs.

## Methods

### Establishment and treatment of animal model

Healthy male C57BL/6J mice, aged six weeks and weighing approximately 18–20 g, were purchased from specific pathogen-free (SPF) (Beijing) Biotechnology Co.,Ltd. (Beijing, China). All experimental procedures were performed in accordance with the guidelines of the Ethics Committee of the Zunyi Medical University (approval no. ZMU21-2306-072). The animals were maintained under SPF conditions (humidity 50 ± 10%, 22 to 25 °C, 12 h light/12 h dark cycle) and allowed free access to water and food.

**Figure 1.**
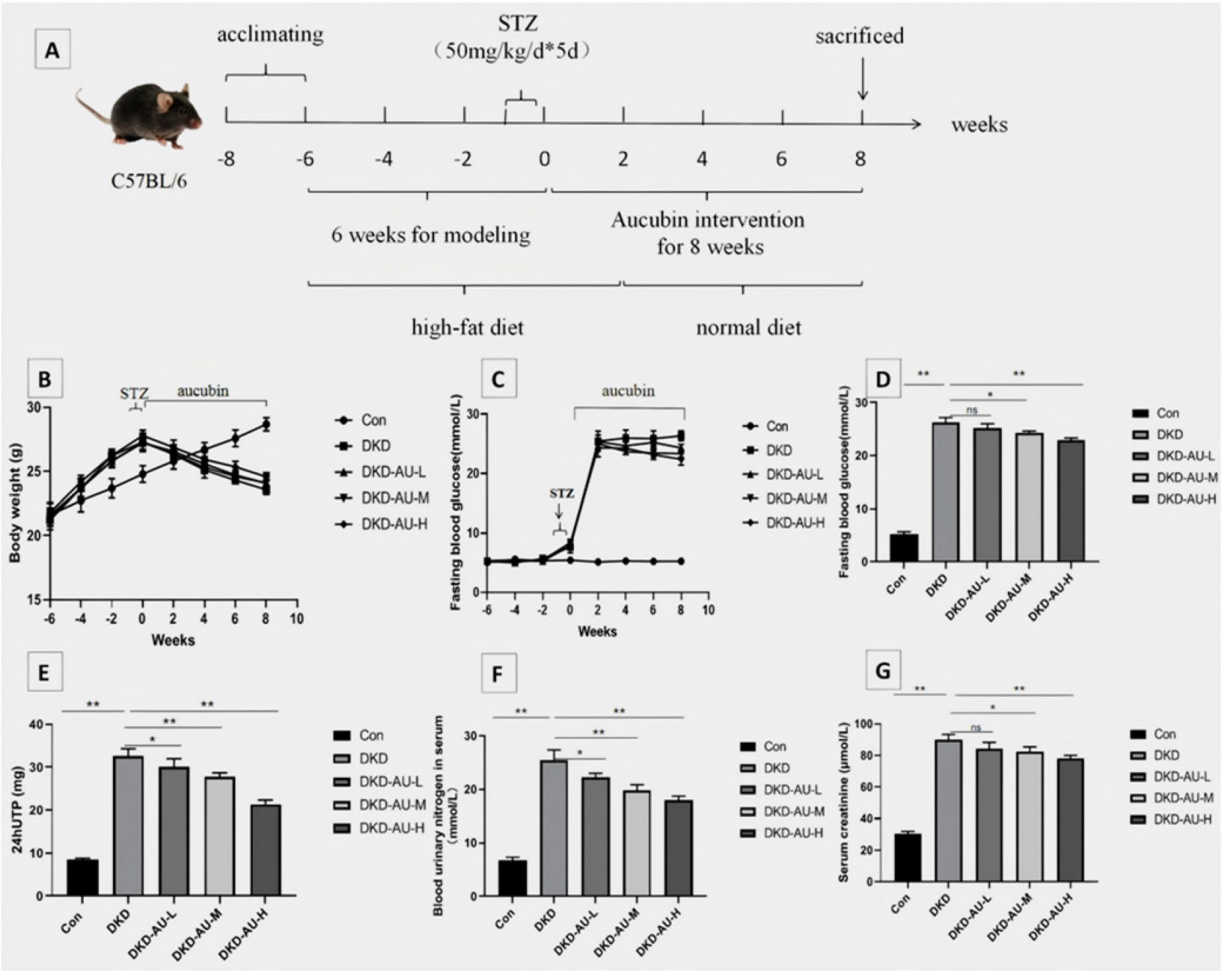
Effects of aucubin on physical and biochemical parameters in DKD mice. (A) The workflow of animal experiments. The changes of (B) body weight and (C) fasting glucose levels for each group at weeks −6, −4, −2, 0, 2, 4, 6, and 8. The changes of (D) fasting glucose, (E) 24hUTP, (F) BUN, and (G) SCr for each group at the end of this experiment. *p < .05; **p < .01. STZ: streptozotocin; DKD: diabetic kidney disease; 24UTP: 24-h urinary total protein; AU: aucubin.

After two weeks of acclimatization, mice were randomly divided into five groups (*n* = 6 for each group): (1) control group (Con), (2) DKD model group (DKD), (3) DKD model + low-dose (5 mg/kg/day) aucubin treatment group (DKD-AU-L), (4) DKD model + middle-dose (10 mg/kg/day) aucubin treatment group (DKD-AU-M), and (5) DKD model + high-dose (15 mg/kg/day) aucubin treatment group (DKD-AU-H). The mice were fed a normal diet in the control group, while the mice were fed a high-fat diet in the first five weeks in the DKD model (20% protein, 20% carbohydrate, 60% fat, total caloric calorie 4.73 kcal/g), followed by streptozotocin (STZ, Sigma Aldrich, USA) intraperitoneal injection (50 mg/kg/d, fasting for 8 h before injection and 4h after injection) for five days. The mice continued to be fed a high-fat diet for 16 days until they changed to a normal diet after developing appetite loss. Both normal and high-fat diets were provided by Beijing Keao Xieli Feed Co.Ltd. Aucubin was purchased from MedChemExpress (HY-N0664; Germany). The mice were intraperitoneally injected with the corresponding concentration of aucubin once daily for eight weeks after modeling. The experimental schedule is shown in Figure 1(A).

The mice were weighed, and random blood glucose (JNJ, Glucose Test Strip) was measured once every two weeks. The 24-h urinary total protein (24UTP) quantification was performed using a test kit provided by the Nanjing Jiancheng Biological Engineering Research Institute (Nanjing, China). After eight weeks of aucubin treatment, mice were anesthetized by intraperitoneal injection of 2% pentobarbital sodium at a dose of 45 mg/kg. Subsequently, 800ul blood was collected from the eyeballs of mice for the detection of creatinine and urea nitrogen using an assay kit (Nanjing Jiancheng Biological Engineering Research Institute). The mice were sacrificed by neck amputation. Both kidneys of mice were removed, and the left kidney tissue was frozen at −80 °C until the detection time of transmission electron microscopy and western blotting (WB), and the right kidney tissue was embedded in paraffin for pathological staining.

### Cell culture and treatment

hGENCs obtained from Procell(CP-H061, Wuhan, China) were cultured in the complete medium (CM-H061, Procell) and maintained at 37 °C in a 5% CO_2_ atmosphere. Cells were treated with different concentrations of aucubin (0, 1, 5, 10, 20, 50, and 100 μM) to determine the safe concentrations that had no obvious effect on cell proliferation and apoptosis. Three doses of aucubin were selected from the safe concentrations to study their protective effects against high glucose damage in hGENCs. In this study, the cells were divided into the following groups: (1) Control group (Con): the cells were cultured in a medium containing 5.6 mM glucose; (2) HG group: the cells were cultured in a medium containing HG (30 mM) [34,35]; (3) HG + low-dose aucubin group (HG AU-L): the cells were treated with 30 mM HG and low-dose aucubin; (4) HG + middle-dose aucubin group (HG AU-M): the cells were cultured in complete medium containing 30 mM glucose and medium-dose aucubin; (5) HG + high-dose aucubin group (HG AU-H), and were cultured in complete medium containing 30 mM glucose and high-dose aucubin. After 24 h of treatment, cell proliferation was detected using cell count kit-8 (CCK-8) and 5-ethynyl-2′-deoxyuridine (EdU) staining, cell apoptosis was detected using flow cytometry, and endothelial mesenchymal transformation (EndMT) markers were detected using immunofluorescence.

### Direct-DIA proteome detection

After 24 h of cultivation, cells from the five experimental groups in Method 2 were collected for further analysis. Cell lysates and protease phosphatase inhibitors were then added to the cells. This step aims to extract total proteins from the cells while preventing protein phosphorylation modifications from being cleaved. The total proteome and phosphoproteome were labeled and quantified using protein spectrometry quantification technology in each group of cells, and the Direct-data-independent acquisition (DIA) method was used for data analysis. Phosphorylated proteins that may be regulated by aucubin and potentially have protective effects on hGENCs against high glucose damage were screened based on the data analysis results, and the total protein and its phosphorylated forms were detected by WB. The WB results were consistent with the trend of the Direct-DIA analysis, which was considered successful. Regulation of the phosphorylation level of this protein will be used as a potential target of aucubin for further experimental studies.

### Plasmid construction and transfection

It has been preliminarily determined that the phosphorylation level of ATG4B at the S383A site may be regulated by aucubin through method 3 study. Three shRNA plasmids, wild-type overexpression plasmids, overexpression plasmids with phosphorylation site mutations, and corresponding control plasmids were constructed by General Biosystems Co., Ltd. (Hefei, China).

The sh-RNA plasmid, sh-ATG4B, was designed to silence the expression of the ATG4B gene and was identified by an endogenous screening method. These can be used to study the effects of reduced ATG4B levels on cellular processes in response to aucubin treatment.

To further determine whether aucubin can exert a protective effect against high glucose-induced damage to hGENCs by regulating the phosphorylation levels of the S383A site of the ATG4B protein, we conducted RNA interference and overexpression experiments targeting the ATG4B gene. The experiment comprised four groups, and different plasmids were transfected into hGENCs: (1) negative control group (NC): transfected negative control plasmid; (2) sh-ATG4B + overexpression control (sh-ATG4B + OVC) group: transfected sh-ATG4B plasmid and overexpressed control plasmid; (3) sh-ATG4B + ATG4B WT group: transfected sh-ATG4B plasmid and wild-type overexpression plasmid; and (4) sh-ATG4B + ATG4B S383A group: transfected sh-ATG4B plasmid and ATG4B S383A mutant plasmid. The cells from each of the four groups were cultured in high-glucose medium and in medium containing high glucose and 20 μM aucubin, respectively.

hGENCs were inoculated into six-well plates for 24h before transfection, and the confluence of cells per well was 60–80%. In the three groups transfected with two plasmids, the first plasmid was transfected in an incubator set at 37 °C for 36 h. During this period, the culture medium was changed at 24 h after transfection; the second plasmid was transfected into the cells using a similar transfection method as the first plasmid. Multiple protein expression levels and changes in cell function were examined.

### Assessment of cell viability

Cell proliferation was assessed using CCK-8 and EdU assays. To perform the CCK-8 assay, cells were seeded at 1 × 10^4^ cells/well in a 96-well plate and incubated for 24h. The CCK-8 reagent (Beyotime, Shanghai, China) was added to the cells and incubated for 2 h. Cell viability was assessed by immediately measuring the absorbance at 450 nm. For the EdU assay, cells were seeded in 24-well plates at a density of 3× × 104 cells/well. After the cells were treated with the respective experimental conditions and incubated for 24 h, the EdU working solution was co-cultured with the cells for 2 h. Subsequent fixation and staining were performed according to the instructions of EdU (Abbkine, Wuhan, China) and photographed using a fluorescent microscope.

### Apoptosis detection

For the cell apoptosis assay, cells were plated at a density of 1 × 10^5^ cells/well in 12-well plates. Cells were treated according to the requirements of each group and incubated for 24h. The apoptotic rate was determined using Annexin V-FITC/APC (MultiSciences, Hangzhou-China) and DAPI on a FACSCalibur flow cytometer (ACEA NovoCyte, USA) and analyzed using FlowJo software.

### Immunofluorescence detection

The cells from each group were incubated in cell culture plates, washed lightly with phosphate buffered saline (PBS) after 24h, fixed with 4% paraformaldehyde for 15 min, washed three times with PBS for 5 min each, and blocked with 5% bovine serum albumin (BSA) for 1 h. Thereafter, the cells were incubated with the diluted primary antibody (1:200 for CD31 and Vimentin) in 1% BSA overnight at 4 °C. After gentle washing with PBS, the cells were incubated with the secondary antibody at room temperature for 1h. Finally, the cells were stained with DAPI for 15 min. The IF images were collected using an Olympus fluorescence microscope and analyzed using ImageJ software.

### Western blot analyses

Cells and renal cortex lysates were homogenized in RIPA lysis buffer containing a protease inhibitor cocktail. Protein concentrations were determined using the bicinchoninic acid assay (BCA). Total protein (20 µg) from each sample was loaded and electrophoretically separated on a sodium dodecyl sulfate polyacrylamide gel. Proteins were transferred to polyvinylidene difluoride membranes (PVDF) and then incubated with specific primary antibodies against ATG4B, p-ATG4B(Ser383), ATG5, ATG7 (1:1,000, CST); Bcl-2, Bax (1:500, Immunoway); p62, cleaved caspase3 (1:500, Immunoway); LC3 (1:1,000, Abcam); CD31, Vimentin (1:2,000, Proteintech); nephrin (1:1,000, Affinity) and α-SMA, GAPDH(1:1,000, Proteintech), and secondary antibodies conjugated to HRP (Proteintech). The bands on the membrane were visualized using Enhanced Chemiluminescence (ECL) and analyzed using ImageJ software.

### Pathological observation of kidney tissues

Kidney tissue was fixed with 10% formaldehyde and embedded in paraffin. To observe pathological changes in the kidneys, 4 µm paraffin-embedded sections were stained with hematoxylin and eosin (HE) and periodic acid–Schiff (PAS).

### Electron microscopy

For electron microscopy, the renal tissue of mice in each group was cut into small pieces of approximately 1mm^3^, fixed with 2.5% glutaraldehyde and 1% osmic acid, rinsed with 0.1 M phosphoric acid bleach solution, fixed with 50%, 70%, 90%, and 100% acetone successively, embedded with embedded solution, sliced into 50–60 nm slices using a microtome, and stained with 3% uranium-lead citrate double. Images were observed and photographed using a JEM-1200EX transmission electron microscope.

### Blinding

To reduce the occurrence of selective bias, we adopted the blinding principle in the interpretation of histological scores and biochemical analyses.

### Statistical analysis

Values are shown as mean ± standard deviation. The data were analyzed using GraphPad Prism 8 Project software. One-way analysis of variance (ANOVA) was used for multiple group comparisons. Statistical analysis was performed using the SPSS Statistics software (version 29.0; IBM Corp., Armonk, NY, USA). *p <* .05.

## Results

### Effect of aucubin on physical and biochemical parameters in DKD mice

Compared with the control group fed with a normal diet, the high-fat diet/STZ mice showed significant weight loss, a marked increase in blood glucose, obvious proteinuria and renal function impairment (Figure 1(B–G)), suggesting that we have successfully established the DKD mouse model [36]. After being treated with different concentrations of aucubin, DKD mice showed a downward trend in blood glucose, a significant reduction in proteinuria, and a alleviation of renal function impairment, especially in the DKD-AU-H group. These results suggest that aucubin has a protective effect on DKD mice (Figure 1(B–G)).

### Effect of aucubin on renal structure in DKD mice

**Figure 2.**
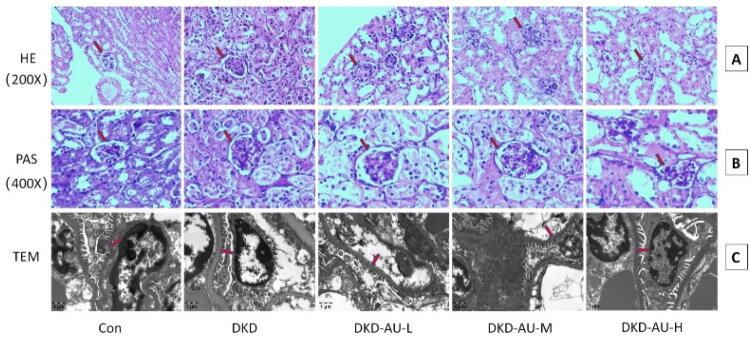
Effects of aucubin on structure of renal tissue in DKD mice. (A) HE: HE staining images, original magnifications of 200×. (B) PAS: PAS staining images, original magnifications of 400×. (C) TEM: transmission electron microscope images, the bar in the lower left represents 1 μm. The red arrows indicate glomerular basement membrane. DKD: diabetic kidney disease; AU: aucubin; HE: hematoxylin-eosin; PAS: periodic acid–Schiff; TEM: transmission electron microscope.

After eight weeks of aucubin intervention, kidney tissues were collected and analyzed. There was no significant difference in kidney weight among the groups. However, histological analysis revealed that the DKD group exhibited mesangial matrix hyperplasia and widened mesangial regions in the glomeruli compared with the control group. These pathological changes were less pronounced in the DKD-AU-L, DKD-AU-M, and DKD-AU-H groups, particularly in the DKD-AU-H group (Figure 2(A,B)). Electron microscopy images showed that the thickness of the glomerular basement membrane was uniform, and the arrangement of foot process cells was orderly in the control group. In contrast, the DKD group showed significantly widened and merged foot processes, and uneven thickening of the glomerular basement membrane. These pathological changes were mitigated in the DKD-AU-L, DKD-AU-M, and DKD-AU-H groups, particularly in the DKD-AU-H group (Figure 2(C)).

### Protective effect of aucubin on high glucose-induced hGENCs injury

**Figure 3.**
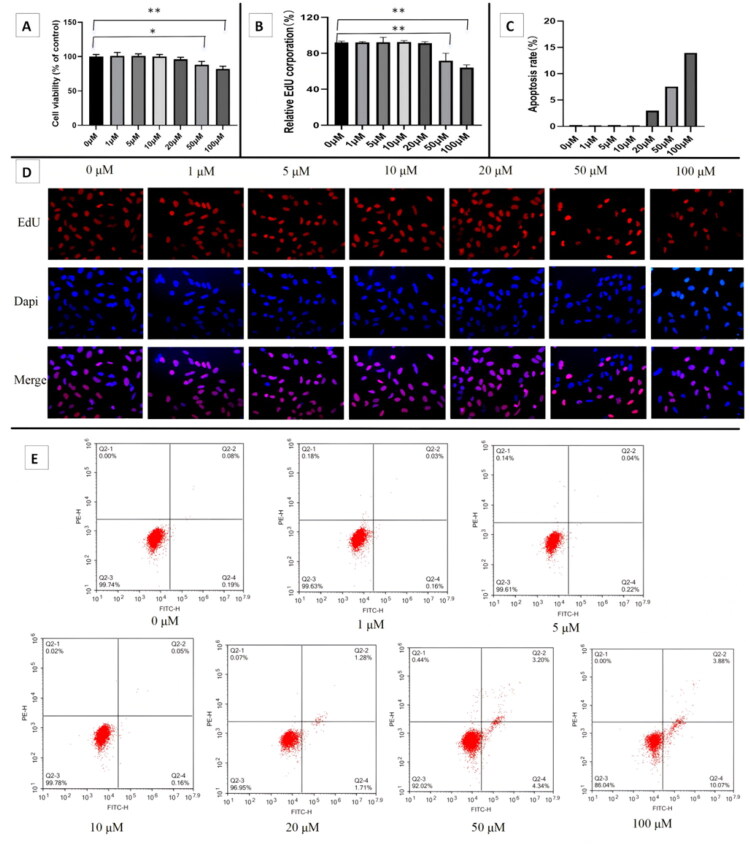
Effects of different concentrations of aucubin on proliferation and apoptosis of hGENCs. (A) Cell proliferation was detected by CCK-8 assay. (B,D) Cell proliferation was detected by EdU staining. (B) Comparison of positive rate of EdU staining. (C,E) Apoptosis was detected by flow cytometry. *p < .05; **p < .01. FITC-H: fluoresceine isothiocyanate-Height; PE-H: phycoerythrin-Height.

**Figure 4.**
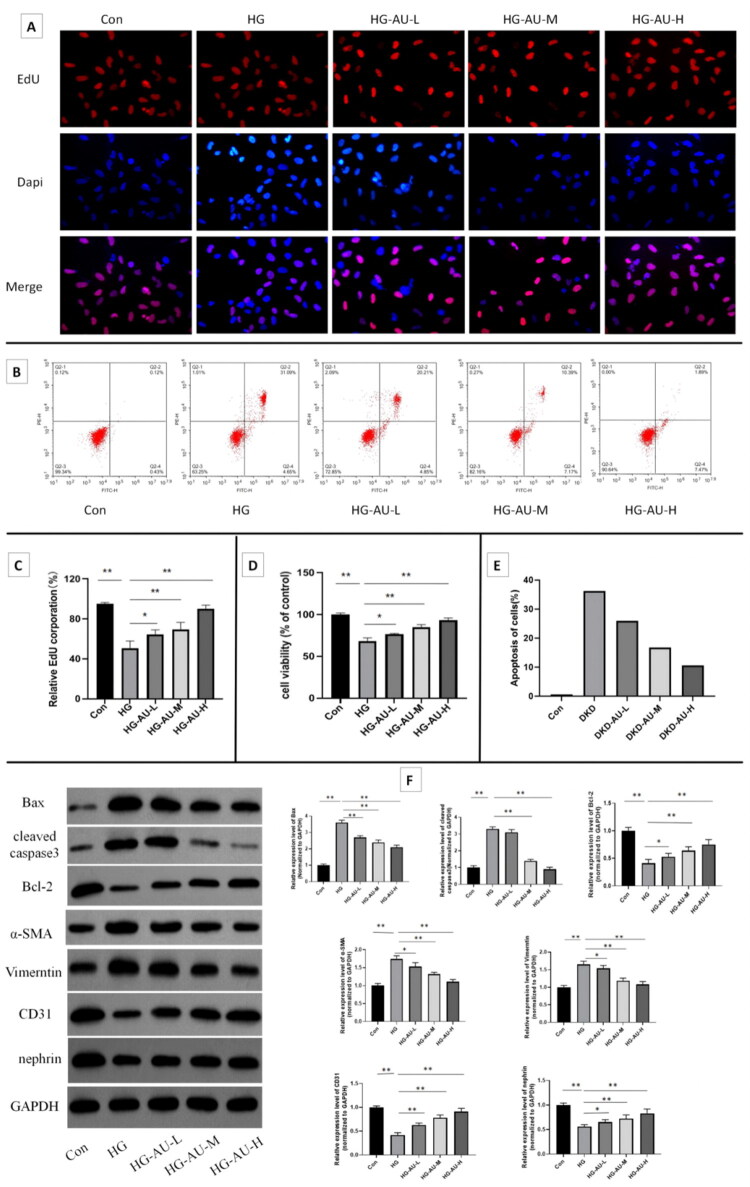
Protective effect of aucubin on high glucose-induced hGENCs injury. (A,C) Cell proliferation detected by EdU staining. (B, E) Apoptosis images were detected by flow cytometry. (D) Cell proliferation was detected by CCK-8 assay. (F) The protein expression levels detected by WB. *p < .05; **p < .01. DKD: diabetic kidney disease; AU: aucubin; HG: high glucose.

hGENCs were treated with various concentrations of aucubin for 24h, respectively. Subsequently, cell viability was detected by the CCK-8 assay and EdU staining, and cell apoptosis was detected by flow cytometry. The results indicated that concentrations of 20 μM or lower had no significant effect on cell proliferation or apoptosis (Figure 3(A–E)). Therefore, in the subsequent experiments, aucubin at concentrations of 5 μM, 10 μM and 20 μM were used to study their protective effects on hGENCs injury induced by high glucose. The results showed that compared with the Con group, under high glucose culture, cell proliferation was significantly weakened, apoptosis was significantly increased, and EndMT occurred in the cells, suggesting that high glucose culture caused damage to hGENCs cells. However, after treatment with aucubin, it played a protective role against the above damage, especially in the HG-AU-H group (Figure 4(A–F)).

### Aucubin protect hGENCs from high glucose-induced injury by promoting ATG4B protein phosphorylation and increasing autophagy

**Figure 5.**
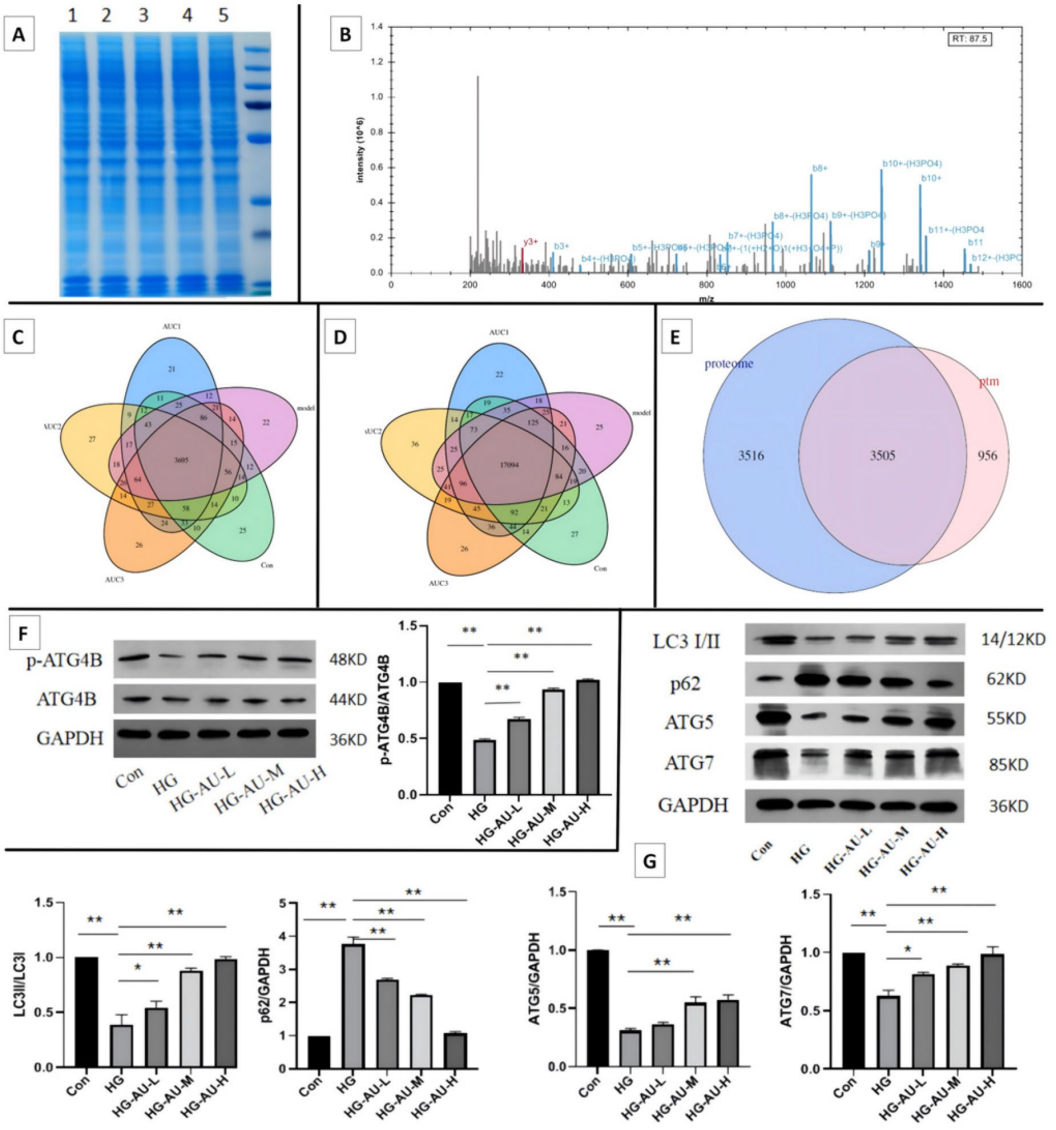
Protective effect of aucubin on high glucose-induced hGENCs injury by promoting ATG4B protein phosphorylation and increasing autophagy. (A) Electrophoretic map of cells in each group. 1, 2, 3, 4, and 5 represent Con group, HG group, HG-AU-L group, HG-AU-M group, and HG-AU-H group, respectively. (B) Mass spectrogram of protein identification. Venn diagram of (C) phosphorylated proteins, (D) phosphorylated protein sites and (E) protein identification, AU1, AU2 and AU3 represent HG-AU-L group, HG-AU-M group, and HG-AU-H group, respectively. (F) Representative images of expression levels of p-ATG4B and ATG4B detected by WB. (G) Representative images of the expression levels of autophagy protein LC3, p62, ATG5, and ATG7 in each group detected by WB, and the statistical analysis result of the proteins expression levels. *p < .05; **p < .01. AU: aucubin; HG: high glucose.

ATG4B is a crucial protein involved in the regulation of autophagy. Direct-DIA analysis revealed that the phosphorylation level (at S383) of ATG4B in the HG group was significantly lower than that in the Con group, and the phosphorylation level (at S383) of ATG4B in the HG-AU-L, HG-AU-M, and HG-AU-H groups was higher than that in the HG group, while there was no significant difference in total protein expression among the five groups. The WB assay further verified that the phosphorylation level of ATG4B and the changing trend of total protein were consistent with the results of Direct-DIA, suggesting that aucubin can increase the phosphorylation of ATG4B (Figure 5(A–F)).

The expression levels of autophagy-related proteins in each group were detected by the WB. The results showed that compared with the Con group, the autophagy function of the cells in the HG group was impaired. After treatment with different concentrations of aucubin, autophagy impairment in hGENCs cultured with high glucose was alleviated, especially in the HG-AU-H group, indicating that aucubin can restore autophagy impairment in hGENCs (Figure 5(G)). These results indicated the protective effect of aucubin on high glucose-induced hGENCs damage by regulating autophagy.

### Aucubin played a protective role in DKD mice by promoting ATG4B protein phosphorylation and regulating autophagy

**Figure 6.**
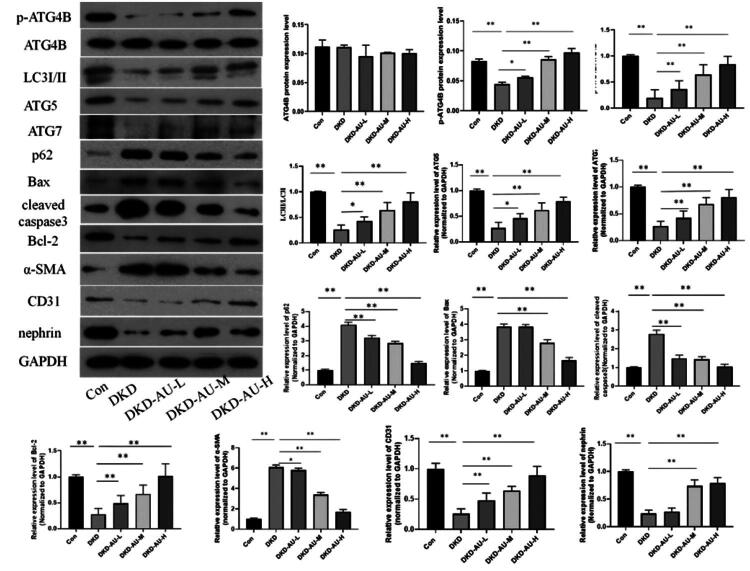
Protective effect of aucubin on DKD mice by promoting ATG4B protein phosphorylation and increasing autophagy. The expression levels of p-ATG4B proteins, ATG4B proteins, autophagy protein (LC3, p62, ATG5, and ATG7), apoptosis-related proteins (Bax, cleaved caspase3, and Bcl-2), EndMT marker proteins (CD31 and α-SMA), and nephrin protein in each group were detected by WB and the statistical analysis result of the proteins expression levels. *p < .05; **p < .01. DKD: diabetic kidney disease; AU: aucubin.

The level of p-ATG4B protein in the DKD group was significantly lower than that in the Con group, and the expression of p-ATG4B in the aucubin-treated groups was significantly higher than that in the DKD group, whereas there was no statistical difference in the expression of ATG4B protein in the kidney tissues of the five groups. The p-ATG4B/ATG4B ratio in the DKD group was significantly lower than that in the Con group, and the ratio in the aucubin-treated groups was higher than that in the DKD group. The WB results also showed that the expression of autophagy-related proteins, apoptosis-related proteins, EndMT marker proteins, and nephrin protein in the kidney tissues of the mouse model was consistent with the cell experiments (Figure 6), suggesting that aucubin played a protective role in DKD mice by promoting ATG4B protein phosphorylation and regulating autophagy.

### Aucubin alleviates high glucose-induced hGENCs injury by restoring autophagy through promoting phosphorylation of ATG4B protein

**Figure 7.**
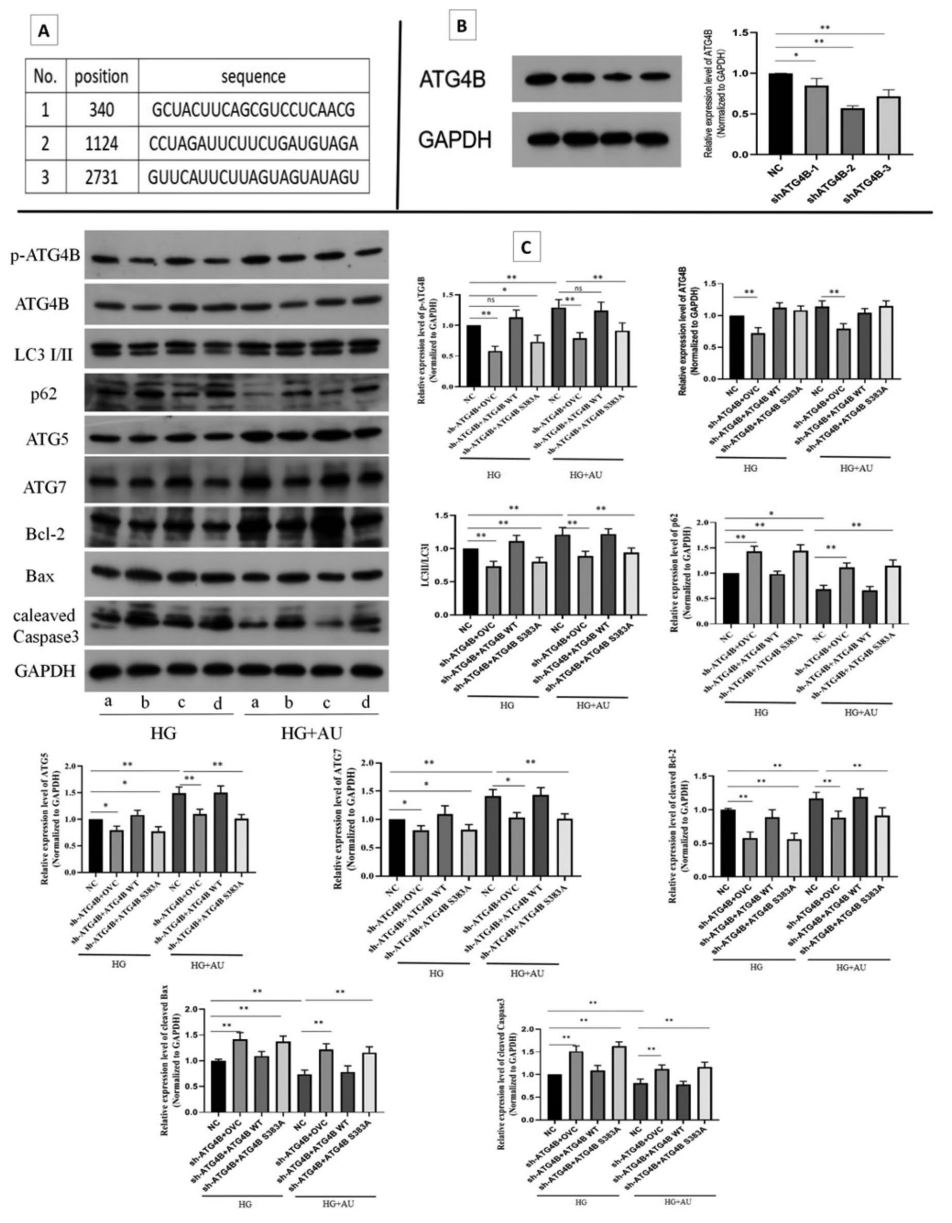
The effect of specific reduction of ATG4B protein phosphorylation levels on related proteins expression in hGENCs under high glucose culture condition and high glucose + aucubin culture condition. (A) Three sh-ATG4B sequences. (B) ATG4B protein expression level was detected by WB after transfection of three sh-ATG4B plasmids into hGENCs. (C) The multiple proteins expression levels detected by WB. a, b, c, and d represent NC group, sh-ATG4B + OVC group, sh-ATG4B + ATG4B WT group, and sh-ATG4B + ATG4B S383A group, respectively. *p < .05; **p < .01. AU: aucubin; HG: high glucose.

The WB results showed that sh-ATG4B-2 had the best inhibitory effect on the expression level of ATG4B (Figure 7(A,B)); therefore, the plasmids containing this sequence were used for following experiments.

**Figure 8.**
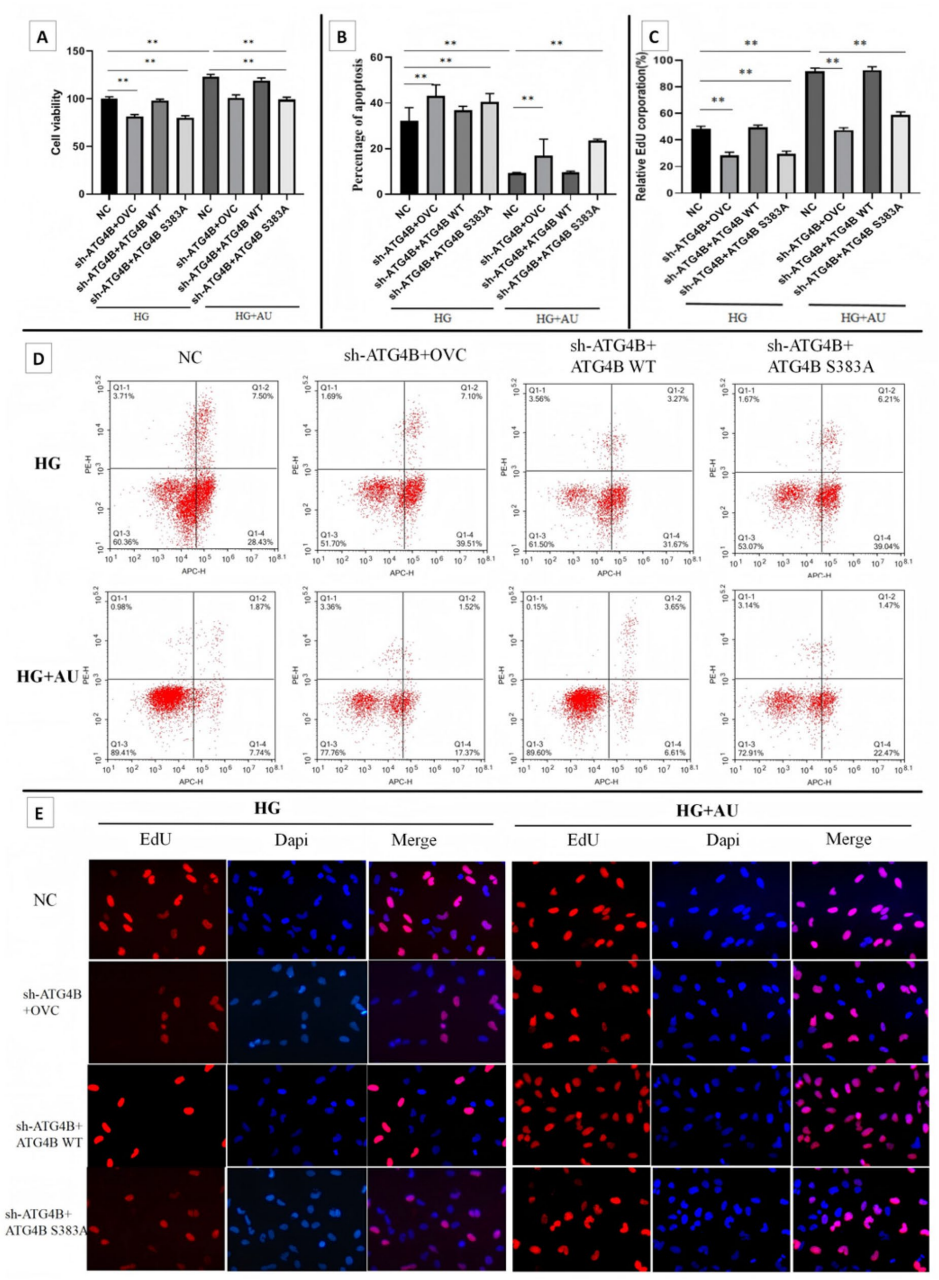
The effect of specific reduction of ATG4B protein phosphorylation levels on proliferation and apoptosis of hGENCs. (A) Cell proliferation was detected by CCK-8 assay. (B, D) The apoptosis were detected by flow cytometry. (C, E) Cell proliferation were detected by EdU staining. **p < .01. AU: aucubin; HG: high glucose; NC: negative control.

To investigate whether aucubin exerts protective effect by regulating the phosphorylation level of the S383A site of the ATG4B protein, the WB were performed and results showed that the level of ATG4B and p-ATG4B proteins in each group were in line with expectations, suggesting that the transfection experiments were successful (Figure 7(C)). Further results indicated that the specific reduction of p-ATG4B protein abundance aggravated HG-induced hGENCs injury, while overexpression of ATG4B WT could reverse this effect. Under high glucose culture, the specific decrease in p-ATG4B protein level promoted EndMT, reduced cell proliferation and increased cell apoptosis. Furthermore, under treating with aucubin in high glucose cultured cells, the specific reduction of p-ATG4B protein abundance eliminated the protective effect of aucubin (Figures 7(C) and 8(A–E)).

## Discussion

Impaired autophagy leads to damage of various types of renal cells, including podocytes, mesangial cells, renal proximal tubular epithelial cells, and glomerular endothelial cells, resulting in the occurrence and progression of DKD. In a mouse model of diabetic kidney disease, TRPC6 mediates the activation of calprotease, leading to impaired autophagy in podocytes, secondary cell damage, proteinuria, and accelerating the progression of DKD [15]. Glomerular mesangial cell senescence induced by advanced glycation end products (AGEs) is accelerated by inhibiting autophagy and induces renal inflammation and fibrosis in DKD [16,37]. Renal proximal tubular epithelial cell (PTEC) lesions are an important factor in the occurrence and progression of diabetic nephropathy. Autophagy activity decreased in mice with DKD and HG-induced human proximal tubular cell injury models [17]. In the model of glomerular endothelial cell injury induced by high glucose, autophagy inducers inhibit inflammation and ROS levels, while autophagy inhibitors have the opposite effect. The study also found that AIF-1 regulates inflammation, oxidative stress and autophagy levels in hrgec through the miR-34a/ATG4B pathway. Participate in the pathogenesis of diabetic nephropathy [18].

Restoring autophagy and maintaining cellular homeostasis have demonstrated potential value in the treatment of various diseases, including DKD. Traditional Chinese medicine or its extracts have promising application prospects in restoring autophagy in cells. The famous traditional Chinese medicine formula, Shao Yao Gan Cao Decoction, protects against APAP-induced liver injury by promoting autophagy/mitophagy [38]. In both *in vitro* and *in vivo* MPP+/MPTP-induced Parkinson’s disease models, Artemisia annua leaf extract protects against neuronal toxicity by activating TRPML1 and promoting autophagy/mitophagy clearance [39]. The extract of red peony root inhibits ferroapoptosis, activates autophagy and alleviates cerebral ischemic injury through the PI3K/Akt signaling pathway [40]. Suoquan Yishen Formula promotes autophagy in diabetic nephropathy and improves renal cell senescence by inhibiting the YTHDF1-Rubicon axis [41]. Triptolide targets autophagy through the mTOR/Twist1 pathway and inhibits the epithelial-mesenchymal transition of podocytes in diabetic nephropathy [42].

Endothelial-mesenchymal transition (EndMT) is the main mechanism of organ fibrosis. Endothelial cell dysfunction and EndMT can promote the progression of DKD, while restoring endothelial cell characteristics. Inhibiting EndMT can prevent DKD [21,43]. It is worth noting that restoring autophagy through various methods has been proven to alleviate EndMT and prevent organ dysfunction [21,44,45].

Our research results indicate that in both the DKD mouse model and the HG-induced human glomerular endothelial cell injury model, aucubin extracted from the traditional Chinese medicine *E. ulmoides* can exert a protective effect by controlling EndMT. Protein phosphorylation was an important form of epigenetic modification that activates functional activation [46–48]. In the mechanistic study, mass spectrometry was employed to conduct whole proteomic + phosphorylated proteomic detection, and direct DIA analysis technology was combined with cell samples from each experimental group. Based on this, we observed significant alterations in ATG4B phosphorylation, a key protein in autophagy regulation, in the cells of each group, while the expression of total protein remained unchanged, suggesting that regulating ATG4B phosphorylation may be a potential target for aucubin. Therefore, we speculated that aucubin might restore autophagy and reduce EndMT by upregulating the phosphorylation level of ATG4B, thus playing a protective role in DKD.

Subsequently, in both *in vivo* and *in vitro* models of DKD, compared with the Con group, the phosphorylation level of ATG4B in the model group was significantly downregulated, and the autophagy ability was significantly decreased. After treatment with aucubin, the phosphorylation level of ATG4B increased significantly, and the autophagy ability recovered. These results are consistent with our expectations. Finally, we performed transfection experiments on hGENCs using RNA interference and gene overexpression. The results showed that specific deletion of p-ATG4B impaired autophagy, promoted EndMT, reduced cell proliferation, increased apoptosis, and exacerbated HG-induced damage to hGENCs. Moreover, the specific deletion of p-ATG4B abolished the aucubin-mediated protective effect against HG-induced hGENCs damage.

Previous studies have reported that the change in total ATG4B protein levels can regulate autophagy and the process of DKD [18,49,50]. In this study, our results showed that a decrease in ATG4B phosphorylation can weaken autophagy function and promote the progression of DKD, whereas aucubin can counter these effects by increasing ATG4B phosphorylation, restoring autophagy, and protecting against DKD.

## Strengths and limitations

Although we have made certain innovative discoveries in the protection of DKD by aucubin, providing some theoretical basis for the treatment of DKD with aucubin, the mechanism by which aucubin achieve their function, such as regulating which phosphorylated kinases, remains unclear and requires further in-depth research to reveal. Regarding whether aucubin can be clinically applied in the treatment of DKD patients in the future, studies involving aspects such as bioavailability, pharmacokinetics and safety also require a large number of subsequent studies to provide support.

## Conclusion

In summary, our data demonstrated that reduced phosphorylation of ATG4B could damage the autophagy ability of cells, promote the occurrence of EndMT, and then participate in the progression of DKD, while aucubin upregulates the phosphorylation of ATG4B, restores autophagy, weakens EndMT, and inhibits the progression of DKD. The regulation of ATG4B phosphorylation is a new potential target for DKD therapy.

## Data Availability

The datasets analyzed during the research are available upon reasonable request from the corresponding author.
